# Correction: Theory of Planned Behavior applied to the choice of food with preservatives by owners and for their dogs

**DOI:** 10.1371/journal.pone.0306288

**Published:** 2024-06-25

**Authors:** Vivian Pedrinelli, Alexandre Rossi, Marcio A. Brunetto

The third author, Marcio A. Brunetto, is incorrectly noted as the corresponding author. The correct corresponding author is Vivian Pedrinelli. Vivian Pedrinelli’s email address is: vivian.pedrinelli@gmail.com.

In the Acknowledgments section, the following information is missing: The authors would like to express their gratitude to Grandfood Industria e Comercio LTDA (PremieRpet^®^) for covering the publication costs.

The updated Acknowledgments section is as follows: Marcio A. Brunetto passed away before the submission of the final version of this manuscript. Vivian Pedrinelli accepts responsibility for the integrity and validity of the data collected and analyzed. The authors would like to express their gratitude to Grandfood Industria e Comercio LTDA (PremieRpet^®^) for covering the publication costs.
