# Supplementary material for: Theory of Planned Behavior applied to the choice of food with preservatives by owners and for their dogs
Source: PLoS One. 2024 Jan 19;19(1):e0294044. doi: 10.1371/journal.pone.0294044 (PMC10798483; doi:10.1371/journal.pone.0294044)
Supplement: S1 File — (DOCX) [file pone.0294044.s001.docx]

**S1 Appendix. Questionnaire**

Section one

1) Select your state of residence:

(All Brazilian states given as options)

2) In what type of household do you live?

a) House

b) Apartment

c) Country house or small farm

d) Other

3) What is your household’s Monthly income?

a) Up to R$ 6,060

b) From R$ 6,6061 to 12,120

c) More than R$ 12,121

d) Do not want to disclose

4) What is your scholarity level?

a) No formal instruction or illiterate

b) Did not complete middle school

c) Completed middle school

d) Did not complete high school

e) Completed high school

f) Did not complet graduate school

g) Completed graduate school or post-graduation

5) What is your age?

a) From 18 to 24 years

b) From 25 to 34 years

c) From 35 to 44 years

d) From 45 to 54 years

e) From 55 to 64 years

f) 65 years or older

6) What is your gender?

a) Female

b) Male

c) Other: ______________

d) Do not want to disclose

7) Do you work at the pet food industry?

a) Yes

b) No

8) Are you a veterinarian or an animal scientist?

a) Yes

b) No

Section two

9) How many dogs do you have?

a) Only one

b) Two or three

c) More than three

In case you have more than one dog, please have in mind for the next questions only one of your dogs (and consider the same dog for all the answers).

10) What is your dog’s breed?

a) Mixed breed

b) Specific breed: _________________

11) How Much does your dog weigh?

a) Up to 6.5 kg

b) From 6.5 kg to 9 kg

c) From 9 kg to 15 kg

d) From 15 kg to 30 kg

e) From 30 kg to 40 kg

f) More than 40 kg

12) What is the gender of your dog?

a) Female

b) Male

13) Is your dog neutered?

a) Yes

b) No

14) Where is your dog allowed to go in your household?

a) All the rooms

b) Some of the rooms

c) Only kept outdoors

15) Where do you search for information on pet food?

a) My veterinarian or technician

b) Friends or family

c) Social media

d) Manufacturer’s website

e) Other websites and blogs

f) Other sources

16) What kind of dog food do you offer as the main meal?

a) Dry kibble diet

b) Wet diet (pouch or can, for example)

c) Homecooked diet

d) Raw diet (BARF, meaty bones and/or prey model, for example)

e) Other:______________

Section three

(5-point Likert scale – completely disagree, partially disagree, indifferent, partially agree and completely agree)

1) I believe that buying food without preservative for my dog will be good.

2) I believe that buying food without preservatives is necessary to the health of my dog.

3) I believe that if my dog eats foods with preservatives, he will be sick

4) I do not buy foods with preservatives for my dog because it is not healthy.

5) I trust in manufacturers of dog food.

6) I can trust in products without preservatives for dogs.

7) I believe in the information stated on the label of my dog’s food.

8) I consider myself a person concerned about my dog’s nutrition.

9) I know people that would like me to buy food without preservatives for my dog.

10) My veterinarian believes that I should buy food without preservatives for my dog.

11) My family members believe I should buy food without preservatives for my dog.

12) I feel that I should buy food without preservatives for my dog’s health.

13) I have the financial resources to buy food without preservatives for my dog.

14) I have the opportunity to buy food without preservatives for my dog.

15) I have the knowledge to buy food without preservatives for my dog.

16) I think of buying food without preservatives for my dog.

17) I will buy food without preservatives for my dog.

18) I often buy food without preservatives for my dog.

19) I buy and have been buying food without preservatives for my dog.

Section four

(5-point Likert scale – completely disagree, partially disagree, indifferent, partially agree and completely agree)

1) I believe that buying food without preservative for myself will be good.

2) I believe that buying food without preservatives is necessary to my health.

3) I believe that if I eat foods with preservatives, I will be sick

4) I do not buy foods with preservatives for myself because it is not healthy.

5) I trust in manufacturers of human food.

6) I can trust in products without preservatives for humans.

7) I believe in the information stated on the label of my food.

8) I consider myself a person concerned about my nutrition.

9) I know people that would like me to buy food without preservatives for myself.

10) My physician believes that I should buy food without preservatives for myself.

11) My family members believe I should buy food without preservatives for myself.

12) I feel that I should buy food without preservatives for my health.

13) I have the financial resources to buy food without preservatives for myself.

14) I have the opportunity to buy food without preservatives for myself.

15) I have the knowledge to buy food without preservatives for myself.

16) I think of buying food without preservatives for myself.

17) I will buy food without preservatives for myself.

18) I often buy food without preservatives for myself.

19) I buy and have been buying food without preservatives for myself.

The affirmations from sections three and four were elaborated and divided into groups and subgroups of direct and indirect measures by the researchers, as stated below:

a) Questions 1 and 2 were for “Attitude”;

b) Questions 3 and 4 were for “Behavioral believes”, subgroup “Health”;

c) Questions 5, 6 and 7 were for “Behavioral believes”, subgroup “Trust in labels and manufacturers”;

d) Question 8 was for “Behavioral believes”, subgroup “Self-identity”;

e) Question 9 was for “Subjective norms”;

f) Questions 10 and 11 were for “Normative believes”, subgroup “Influence of others”;

g) Question 12 was for “Normative believes”, subgroup “Personal influence”;

h) Questions 13, 14 and 15 were for “Perceived behavioral control”;

i) Questions 16 and 17 were for “Intention”;

j) Questions 18 and 19 were for “Behavior”.

The owners did not have access of the grouping of the questions.
