# Supplementary material for: Theory of Planned Behavior applied to the choice of food with preservatives by owners and for their dogs
Source: PLoS One. 2024 Jan 19;19(1):e0294044. doi: 10.1371/journal.pone.0294044 (PMC10798483; doi:10.1371/journal.pone.0294044)
Supplement: S1 Table — (DOCX) [file pone.0294044.s002.docx]

**S1 Table. Factor loadings**

|  | **Dogs** | **Owner** |
| --- | --- | --- |
| **Attitude** |  |  |
| ***Intention*** | 0,428 | 0,368 |
| **Subjective norms** |  |  |
| ***Intention*** | 0,135 | 0,076 |
| **Perceived behavioral control** |  |  |
| ***Intention*** | 0,401 | 0,492 |
| ***Behavior*** | 0,337 | 0,291 |
| **Intention** |  |  |
| ***Behavior*** | 0,337 | 0,529 |
