# Supplementary material for: Theory of Planned Behavior applied to the choice of food with preservatives by owners and for their dogs
Source: PLoS One. 2024 Jan 19;19(1):e0294044. doi: 10.1371/journal.pone.0294044 (PMC10798483; doi:10.1371/journal.pone.0294044)
Supplement: S2 Table — (DOCX) [file pone.0294044.s003.docx]

**S2 Table. Model fit**

|  | **Dogs** | **Owner** |
| --- | --- | --- |
| **CFI** | 0,990 | 1,000 |
| **GFI** | 0,991 | 0,998 |
| **AGFI** | 0,935 | 0,998 |
| **TLI** | 0,952 | 1,003 |
| **RMSEA** | 0,101 | 0,022 |
| **SRMR** | 0,0194 | 0,003 |

Legend: CFI = Comparative Fit Analysis; GFI = Goodness of Fit Index; AGFI = Adjusted Goodness of Fit Index; TLI = Tucker Lewis Index; RMSEA = Root Mean Square Error of Approximation; SRMR = Standardized Root Mean Square Residual.
