# Supplementary material for: Theory of Planned Behavior applied to the choice of food with preservatives by owners and for their dogs
Source: PLoS One. 2024 Jan 19;19(1):e0294044. doi: 10.1371/journal.pone.0294044 (PMC10798483; doi:10.1371/journal.pone.0294044)
Supplement: Attachment — Submitted filename: Response to reviewers - TCP.docx [file pone.0294044.s004.docx]

Response to reviewers

ACADEMIC EDITOR:

 Authors are advised to test for the existence of common method bias in the dataset given that there is high probability of receiving socially desirably answers from the respondents. Provide all the relevant results from the analysis including the measurement model results, the model fitness and the structural model results. Highlight the implications of the key findings in the concluding part of the paper. I wish you all the best.

Response: Thank you for your comment. We added wo tables in the Supplement section (S2) that include the model fit and factor loadings. We added a description in the Material and Methods section to describe the CFA (lines 135-138) as well as in the Results section (lines 204-207). We also added in the conclusion the highlights of the findings (lines 287-291).

Reviewer 1

The authors exhibit a strong methodological approach which ensures the fitness of the model and reliability of the data used for the study. The source of the scales for measuring the variables used are provided; diagnostic tests such as normality and validity tests were conducted. The authors portray a good understanding of the subject area, existing literature and the Theory of Planned Behaviour employed in explaining their findings. Further, the paper is written in an easy to read way which enhances understandability.

Given that the data was collected using google forms via social media, there is the risk of reaching the wrong audience which could affect the data. Again, using self-reported questionnaires poses the risk of receiving socially desirable answers. However, the authors did not indicate how this risk is mitigated.

The authors should include a section that highlights the various limitations of the study, especially with the data collection process, and how these were addressed.

Response: Thank you for your comments. We added a paragraph with limitations of the study and how they were addressed (lines 276-283).
